# Synthesis, X-ray Diffraction, Thermogravimetric and DFT Analyses of Pyrimidine Derivatives

**DOI:** 10.3390/molecules191117187

**Published:** 2014-10-24

**Authors:** Assem Barakat, Hany J. Al-Najjar, Abdullah M. Al-Majid, Syed F. Adil, Mohamed Ali, Vijay H. Masand, Hazem A Ghabbour, Hoong-Kun Fun

**Affiliations:** 1 Department of Chemistry, College of Science, King Saud University, P.O. Box 2455, Riyadh 11451, Saudi Arabia; 2 Department of Chemistry, Faculty of Science, Alexandria University, P.O. Box 426, Ibrahimia, Alexandria 21321, Egypt; 3 Petrochemical Research Chair, Department of Chemistry, College of Science, King Saud University, B.O. 2455, Riaydh 11451, Saudi Arabia; 4 Department of Chemistry, Vidya Bharati College, Amravati, Maharashtra 444602, India; 5 Department of Pharmaceutical Chemistry, College of Pharmacy, King Saud University, P.O. Box 2457, Riyadh 11451, Saudi Arabia; 6 X-ray Crystallography Unit, School of Physics, Universiti Sains Malaysia, 11800 USM, Penang, Malaysia

**Keywords:** pyrimidine, X-ray, DFT, TGA, FT-IR

## Abstract

An eco-benign synthesis of pyrimidine derivatives **2a**,**b** containing different functional groups with different electronic character starting from nitroalkenes **1a** and **2b** has been described. The structures for **1a** and **2a**,**b** have been characterized by single crystal X-ray diffraction analysis. The thermal data of the molecules pointed towards important structural aspects of their stability. The mechanism of their thermal decomposition is discussed. The thermodynamic parameters of the dissociation steps were evaluated and discussed. DFT calculations reveal that the compound **1a** possesses a high calculated dipole moment value (8.28 D) which indicates its high reactivity towards its surrounding molecules.

## 1. Introduction

Pyrimidine and its derivatives, which are important *N*-heterocyclic molecules, have received the consideration of researchers due to their significant biological and pharmaceutical properties. Pyrimidine and its derivatives have immense importance as antibiotics, and as crucial parts of many vitamins, and coenzymes. Indeed, some of them are the basic constituents of DNA and RNA, and play an important role in the constitutional properties of nucleic acids. Pyrimidine-derived biomolecules have received much attention from spectroscopists, drug, clinical, and industrial researchers because of their therapeutic importance [1,2,3,4,5,6,7,8,9]. Barbiturates (barbituric acid derivatives) are a class of central nervous system depressants, [10] utilized as sedatives, sleeping agents, hypnotics, anxiolytics, anticonvulsants, and anesthetics [7]. In addition, they have additional pharmacological activities as antioxidant anxiolitics, analeptics, anti-AIDA, immunomodulatory, anticancer agents and in other psychiatric disorders, and possess effects on motor and sensory functions [11,12,13,14,15] For example, phenobarbital, a 5-alkylated barbituric acid, was reported to exhibit sedative and hypnotic properties, and most importantly is an anticonvulsant [16]. 

In continuation of our research program [17,18,19,20,21,22,23], in the present work, the structures of some pyrimidine derivatives were characterized for the first time by single-crystal crystallography and TGA studies. DFT calculations were undertaken to study the optimized molecular structural parameters, vibrational frequencies, thermodynamic parameters, total dipole moment and HOMO-LUMO energy gap for the synthesized molecules using B3LYP/6-311G(d,p) basis set. The findings of these spectroscopic and theoretical studies are reported herein.

## 2. Results and Discussion

### 2.1. Synthesis of the Pyrimidine Derivatives 

Green chemistry is being increasingly exploited as a powerful tool for the generation of privileged medicinal scaffolds and fine chemicals due to its numerous advantages, such as providing high enantio- and regioselectivity and more environmentally friendliness. 

**Scheme 1:**
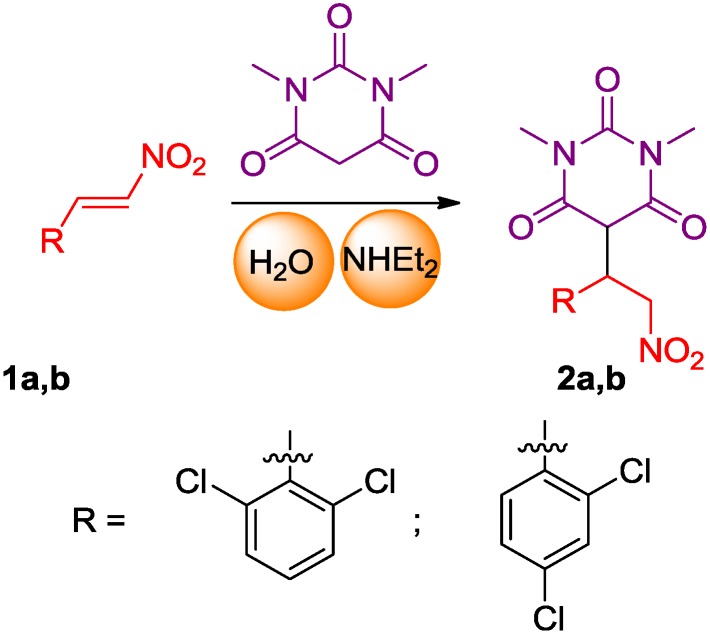
Synthesis of pyrimidine derivatives **2a** and **2b**.

Recently, research groups have become involved in using green chemistry as a synthetic tool for the generation of valuable scaffolds to achieve new biological knowledge. The chemistry used in this paper involves an aqueous diethylamine catalyzed Michael addition of 1,3-dimethylbarbituric acid to nitro-olefins at room temperature for less than 1 h [16] (Scheme 1).

### 2.2. X-ray Crystal Structures

**Figure 1:**
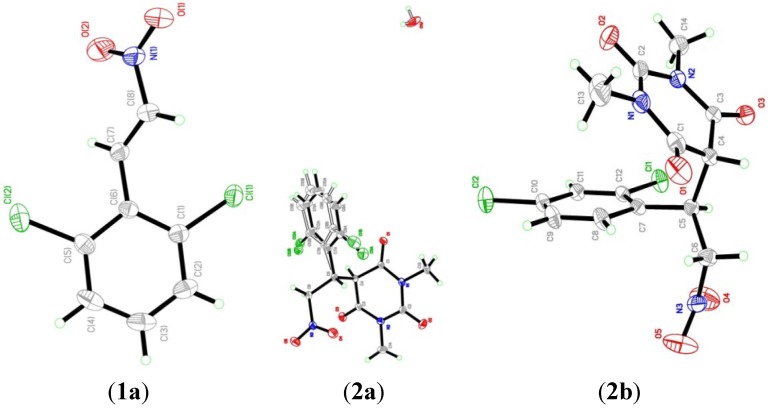
ORTEP diagrams of the structures of **1a** and **2a**,**b**.

The structures of **1a** and **2a**,**b** were confirmed by single crystal X-ray analysis (Figure 1). Table 1, Table 2, Table 3, Table 4, Table 5, Table 6 and Table 7 display the crystal data and main geometrical parameters of the compounds.

X-ray analysis was performed using a Bruker Apex II D8 Venture diffractometer (Bruker AXS GmbH, Karlsruhe, Germany). The data were processed with SAINT and corrected for absorption using SADABS [24]. The structure was solved by direct method using the program SHELXTL [25] and were refined by full-matrix least squares technique on *F*^2^ using anisotropic displacement parameters. The non-hydrogen atoms were refined anisotropically. In these compounds, all the H atoms were calculated geometrically with isotropic displacement parameters set to 1.2 times the equivalent isotropic U values of the parent carbon atoms.

**Figure 2:**
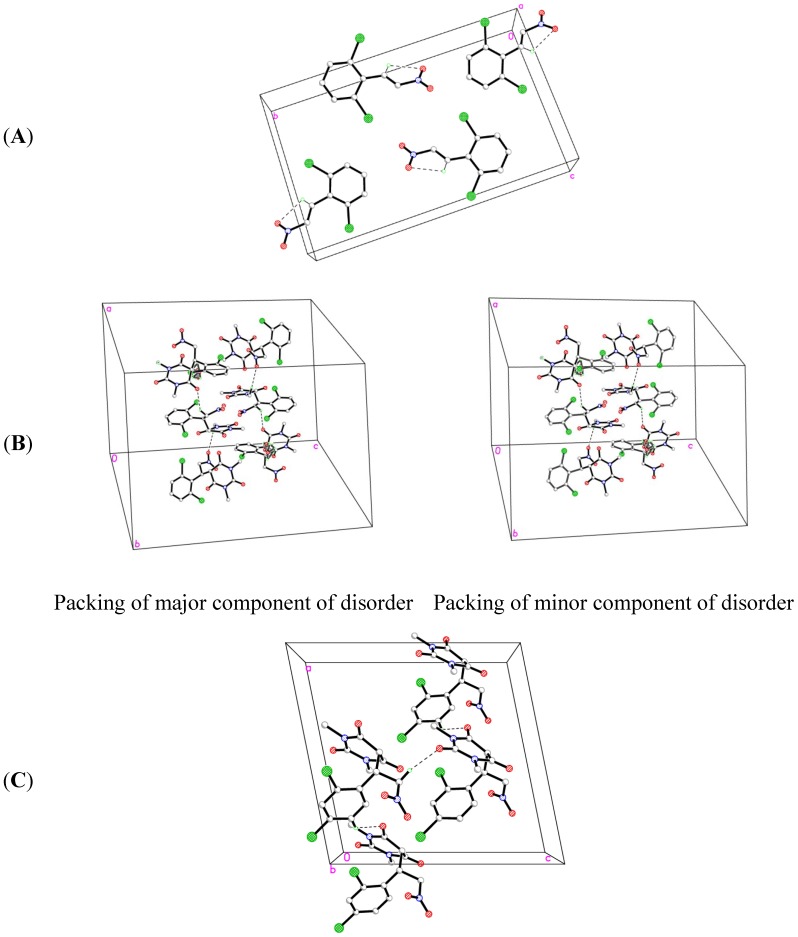
Crystal packings of (**A**) **1a**, (**B**) **2a** and (**C**) **2b**.

The title compound **1a**, C_8_H_5_Cl_2_NO_2_, which crystallizes in the monoclinic space group P 21/c comprises one crystallographically independent molecule in its asymmetric unit, as depicted in Figure 1. In the crystal structure (Figure 2A), there is an intramolecular C7-H7A···O2 hydrogen bond. The crystal is essentially consolidated by Van Der Waals interactions. The crystal data and parameters for structure refinement of the title compound are given in Table 1. Selected geometric parameters are given in Table 2. H-bonding interactions are listed in Table 3.

The title compound **2a**, C_14_H_13_Cl_2_N_3_O_5_·H_2_O, which crystallizes in the trigonal space group R-3 comprises one crystallographically independent molecule with disorder in the phenyl ring and a water molecule in its asymmetric unitas shown in Figure 1. Figure 2B shows the crystal packing for the major and minor components of **2a** respectively with occupancy ratio 0.755:0.245. There are two intermolecular C6-H6A···O2 and C9-H9A···O3 hydrogen bonds (Table 5). The crystal structure is further consolidated by Van Der Waals interactions. The crystal data and parameters for structure refinement of the title compound are given in Table 1. Selected geometric parameters are given in Table 4. H-bonding interactions are listed in Table 5.

The title compound **2b**, C_14_H_13_Cl_2_N_3_O_5_, which crystallizes in the monoclinic space group Cc comprises one crystallographically independent molecule in its asymmetric as shown in Figure 1. In the crystal structure (Figure 2C), there are two intermolecular C6-H6A···O1 and C13-H13C···O5 hydrogen bonds (Table 7). The crystal is further consolidated by Van Der Waals interactions. The crystal data and parameters for structure refinement of the title compound are given in Table 1. Selected geometric parameters are given in Table 6. H-bonding interactions are listed in Table 7.

**Table 1:** Crystal and experimental data of compounds **1a**, **2a** and **2b**.

Parameters	Compound 1a	Compound 2a	Compound 2b
Empirical formula	C_8_H_5_Cl_2_NO_2_	C_14_H_13_Cl_2_N_3_O_5_·H_2_O	C_14_H_13_Cl_2_N_3_O_5_
Formula weight	218.03	391.02	374.17
Temperature	293(2) K	293(2) K	293(2) K
Wavelength (Mo *K*α radiation, λ)	0.71073 Å	0.71073 Å	0.71073 Å
Crystal system	monoclinic	trigonal	Monoclinic
Space group	P 21/c	R-3	Cc
Unit cell dimensions	a = 3.8395 (1) Å, α = 90.00° b = 19.8653 (7) Å β = 90.6258 (10)° c = 11.9619 (4) Å, γ = 90.00°	a = 18.0177(7) Å, α = 90.00° b = 18.0177(7) Å, β = 90.00° c = 26.6558(9) Å, γ = 120.00°	a = 12.9432 (4) Å, α = 90.00° b = 9.3084 (4) Å, β = 101.3572 (16)° c = 13.4650 (5) Å, γ = 90.00°
Volume	912.31 (5)Å^3^	7494.1(6) Å^3^	1590.51 (11) Å^3^
Z	4	6	4
Density (calculated)	1.587 Mg/m^3^	1.535 Mg/m^3^	1.563 Mg/m^3^
Absorption coefficient	0.67 mm^−1^	0.424 mm^−1^	0.439 mm^−1^
*F*(000)	440	3564	768
Crystal size	0.58 × 0.35 × 0.27 mm	0.33 × 0.21 × 0.09 mm	0.57 × 0.35 × 0.26 mm
Theta range for data collection	2.7 to 30.5°.	2.3 to 30.6°.	2.7 to 30.6°.
Index ranges	−5 ≤ h ≤ 5, −28 ≤ k ≤ 28, −17 ≤ l ≤ 17	−25 ≤h ≤ 25, −25 ≤ k ≤ 25, −38 ≤ l ≤ 38	−18 ≤ h ≤ 18, −13 ≤ k ≤ 13, −19 ≤ l ≤ 19
Reflections collected/ unique	41303/2404 [R(int) = 0.042]	107278/5130 [R(int) = 0.059]	35961/4754 [R(int) = 0.024]
Completeness to theta = 30.57°	99.8%	99.6%	99.6%
Absorption correction	multi-scan	multi-scan	multi-scan
Refinement	method Full-matrix least-squares on *F*^2^	method Full-matrix least-squares on *F*^2^	method Full-matrix least-squares on *F*^2^
Goodness-of-fit on *F*^2^	1.04	1.03	1.27
Largest diff. peak and hole	0.28 and −0.22 e.Å^−3^	0.72 and −0.26 e.Å^−3^	0.22 and −0.25 e.Å^−3^

**Table 2:** Selected geometric parameters (Å, °) of compound **1a**.

**Bond**	**Experimental**	**Calculated**
Cl1-C1	1.7320 (13)	1.7314
Cl2-C5	1.7295 (14)	1.7405
O1-N1	1.222 (2)	1.2370
O2-N1	1.211 (2)	1.2372
N1-C8	1.4563 (18)	1.4511
**Atom Angle**	**Experimental**	**Calculated**
O1-N1-O2	124.37 (14)	125.1111
O1-N1-C8	115.73 (14)	115.6492
O2-N1-C8	119.90 (14)	119.2397
Cl1-C1-C2	117.62 (11)	124.7209
Cl1-C1-C6	120.34 (9)	113.6358
Cl2-C5-C4	118.18 (11)	114.3525
Cl2-C5-C6	119.58 (10)	123.4094
N1-C8-C7	120.57 (13)	119.8515

**Table 3:** Hydrogen-bond geometry (Å, °) of compound **1a**.

*D*-H···*A*	*D*-H	H···*A*	*D*···*A*	*D*-H···*A*
C7-H7A···O2	0.9300	2.4000	2.7323 (18)	101.00

**Table 4:** Selected geometric parameters (Å, °) of compound **2a**.

**Bond**	**Experimental**	**Calculated**
Cl1A-C8A	1.738 (4)	1.7277
Cl1B-C8B	1.721 (14)	NC
Cl2A-C12A	1.751 (6)	1.7365
Cl2B-C12B	1.715 (14)	NC
O1-C1	1.204 (2)	1.2278
O2-C2	1.205 (2)	1.2325
O3-C3	1.212 (2)	1.2276
O4-N3	1.196 (3)	1.2362
O5-N3	1.215 (3)	1.2378
O1W-O1W ^i^	0.969 (19)	NC
O1W-O1W ^ii^	0.97 (2)	NC
N1-C1	1.378 (2)	1.3855
N1-C13	1.473 (3)	1.4513
N1-C2	1.382 (2)	1.3724
N2-C2	1.387 (3)	1.3925
N2-C14	1.463 (3)	1.4515
N2-C3	1.370 (2)	1.3881
N3-C6	1.500 (2)	1.4962
**Atom Angle**	**Experimental**	**Calculated**
O1W ^ii^-O1W-O1W ^i^	60.0 (18)	NC
C1-N1-C2	124.36 (15)	122.9525
C2-N1-C13	117.61 (15)	117.6313
C1-N1-C13	117.39 (15)	117.0595
C2-N2-C3	124.74 (15)	123.0628
C3-N2-C14	117.62 (17)	116.9594
C2-N2-C14	117.50 (16)	117.4505
O4-N3-C6	121.03 (18)	117.2445
O4-N3-O5	123.45 (19)	125.7819
O5-N3-C6	115.53 (16)	116.9697
O1-C1-N1	121.69 (16)	123.7575
O1-C1-C4	121.91 (15)	122.9936
N1-C1-C4	116.24 (15)	113.2488
O2-C2-N2	120.68 (18)	123.3871
N1-C2-N2	117.42 (14)	116.2983
O2-C2-N1	121.9 (2)	121.7454
O3-C3-C4	121.55 (15)	123.7560
O3-C3-N2	121.13 (15)	121.7802
N2-C3-C4	117.24 (14)	112.8565
N3-C6-C5	112.43 (14)	111.9825
Cl1A-C8A-C7A	119.7 (3)	124.0885
Cl1A-C8A-C9A	117.4 (3)	114.4806
Cl1B-C8B-C9B	116.7 (10)	NC
Cl1B-C8B-C7B	121.7 (9)	NC
Cl2A-C12A-C7A	120.8 (3)	122.7008
Cl2A-C12A-C11A	114.7 (4)	115.2511
Cl2B-C12B-C7B	122.5 (8)	NC
Cl2B-C12B-C11B	114.4 (10)	NC

Symmetry codes: (i) –*x* + *y* + 1, −*x* + 1, *z*; (ii) –*y* + 1, *x* − *y*, *z*. NC: Not calculated.

**Table 5:** Hydrogen-bond geometry (Å, °) of compound **2a**.

*D*-H···*A*	*D*-H	H···*A*	*D*···*A*	*D*-H···*A*
O1W-H2W1···O1W ^iii^	0.9100	2.1000	2.859 (6)	140.00
O1W-H2W1···O1W ^iv^	0.9100	1.8500	2.689 (6)	152.00
O1W-H2W1···O1W ^v^	0.9100	2.0500	2.689 (7)	125.00
C5-H5A···O4	0.9800	2.2700	2.741 (3)	108.00
C6-H6A···O1 ^vi^	0.9700	2.5300	3.337 (3)	141.00
C6-H6B···Cl2A	0.9700	2.6800	3.125 (4)	109.00
C6-H6B···O3	0.9700	2.5800	2.979 (2)	105.00
C13-H13A···O2	0.9600	2.2900	2.716 (3)	106.00
C13-H13C···O5 ^vii^	0.9600	2.5100	3.425 (3)	159.00
C14-H14B···O2	0.9600	2.2400	2.690 (4)	108.00

Symmetry codes: (iii) −*x* + 4/3, −*y* + 2/3, −*z* + 2/3; (iv) *y* + 1/3, −*x* + *y* + 2/3, −*z* + 2/3; (v) *x* − *y* + 1/3, *x* − 1/3, −*z* + 2/3; (vi) *y* − 1/3, −*x* + *y* + 1/3, −*z* + 4/3; (vii) −*x* + *y*, −*x*, *z*.

**Table 6:** Selected geometric parameters (Å, °) of compound **2b**.

**Bond**	**Experimental**	**Calculated**
Cl1-C12	1.7310 (11)	1.7247
Cl2-C10	1.7338 (13)	1.7195
O1-C1	1.2101 (19)	1.2276
O2-C2	1.219 (2)	1.2325
O3-C3	1.2148 (16)	1.2278
O4-N3	1.212 (2)	1.2362
O5-N3	1.204 (3)	1.2378
N1-C1	1.376 (2)	1.3881
N1-C2	1.386 (2)	1.3925
N1-C13	1.469 (2)	1.4515
N2-C2	1.3947 (19)	1.4071
N2-C3	1.3661 (16)	1.3855
N2-C14	1.471 (2)	1.4513
N3-C6	1.496 (2)	1.4962
**Atom Angle**	**Experimental**	**Calculated**
C1-N1-C2	124.73 (12)	122.7743
C1-N1-C13	117.37 (15)	117.4505
C2-N1-C13	117.89 (15)	117.0006
C2-N2-C3	124.33 (11)	122.9525
C2-N2-C14	117.89 (12)	117.0593
C3-N2-C14	117.64 (12)	117.6313
O4-N3-O5	122.4 (2)	125.7819
O4-N3-C6	118.79 (15)	116.9697
O5-N3-C6	118.86 (17)	117.2445
O1-C1-N1	122.31 (14)	123.3871
O1-C1-C4	120.81 (13)	123.7560
N1-C1-C4	116.80 (12)	112.8565
O2-C2-N1	121.70 (15)	121.7802
O2-C2-N2	120.54 (14)	121.7454
N1-C2-N2	117.74 (12)	116.2983
O3-C3-N2	121.68 (12)	123.7575
O3-C3-C4	121.06 (11)	122.9936
N2-C3-C4	117.18 (10)	113.2488
N3-C6-C5	109.74 (11)	111.9825
Cl2-C10-C9	118.98 (10)	119.8634
Cl2-C10-C11	119.27 (9)	119.9545
Cl1-C12-C7	121.01 (8)	123.6597
Cl1-C12-C11	116.44 (9)	115.4596

**Table 7:** Hydrogen-bond geometry (Å, °) of compound **2b**.

*D*-H···*A*	*D*-H	H···*A*	*D*···*A*	*D*-H···*A*
C6-H6A···O2 ^i^	0.9700	2.3900	3.222 (2)	144.00
C6-H6B···O1	0.9700	2.5500	3.118 (2)	117.00
C9-H9A···O3 ^ii^	0.9300	2.5100	3.2290 (18)	134.00
C13-H13B···O2	0.9600	2.3100	2.727 (3)	105.00
C14-H14C···O2	0.9600	2.2700	2.715 (2)	107.00

Symmetry codes: (i) *x*, −*y* + 1, *z*+1/2; (ii) *x* − 1/2, *y* + 1/2, *z*.

### 2.3. Optimized Molecular Geometry

**Figure 3:**
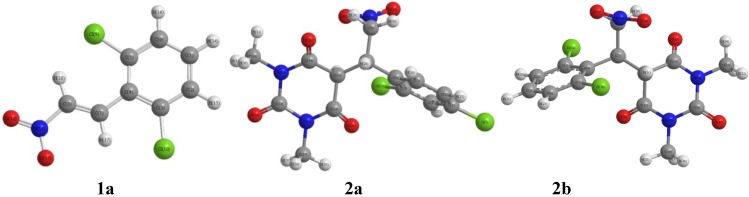
The optimized structures for **1a** and **2a**,**b**.

From the XRD data, it is clear that the compounds **1a** and **2b** possess monoclinic, whereas, **2a** has trigonal crystal structures. The cell dimensions and other data are tabulated in Table 1. Selected values of experimental and DFT calculated geometric parameters for the compounds are listed in Table 2, Table 4, Table 6 and Table 8. Figure 3 shows the optimized structures for **1a** and **2a**,**b**. 

**Table 8:** Optimized calculations of various parameters for **1a** and **2a**,**b** using B3LYP/6-311G basis set.

Parameter	1a	2a	2b
Heat of Formation (kcal/mol)	22.075	−100.903	−99.472
Total Energy (kcal/mol)	−896424.216	−1244859.443	−102495.469
Dipole (Debye)	8.281	4.116	3.848
HOMO energy (eV)	−10.019	−10.575	−10.813
LUMO energy (eV)	−4.320	−2.278	−2.383
HOMO–LUMO energy gap (eV)	5.699	8.297	8.430

**Figure 4:**
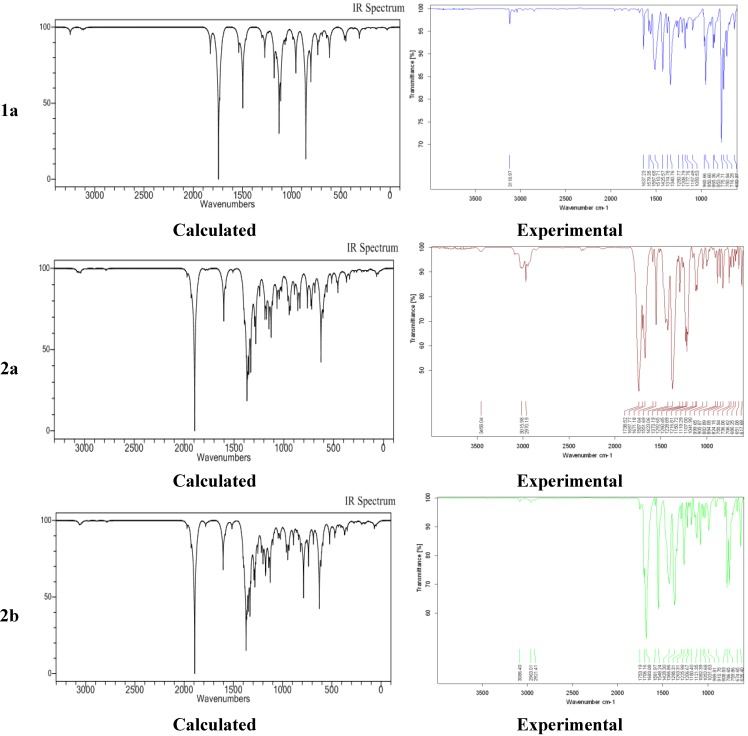
Experimental and calculated IR spectra for **1a** and **2a**,**b**.

Figure 4 shows the experimental and calculated (B3LYP/6-311G) IR spectrum for **1a** and **2a**,**b**. A comparison of predicted (B3LYP/6-311G) with experimental IR reveals that B3LYP/6-311G basis set gives reasonable deviations from the experimental values. The discrepancies observed between the calculated and the experimental vibrational frequencies may be attributable to the fact that the calculations have been actually performed on a single molecule in the gaseous state contrary to the experimental values recorded in the solid state in the presence of intermolecular interactions.

### 2.4. Experimental and Calculated IR Vibrations 

The calculated (B3LYP/6-311G) and experimental C-H, C-Cl, N=O, C=O, and C=C vibration values for all the molecules have good agreement, except for C=O for **2a**. For **2a**, the computed C=O stretching vibrations at 1894 cm^−1^ deviated substantially from the experimental result. A plausible reason for this could be the intermolecular interactions present in solid state whereas during DFT calculations the molecule is assumed to be in isolated gaseous state.

### 2.5. Geometric Parameters

The solid state X-ray structure analysis revealed disorder in the phenyl moiety for **2a** (see Figure 2). This incongruity noted between the calculated and the experimental vibrational frequencies may be due to the fact that the calculations have been actually performed on a single molecule in the gaseous state contrary to the experimental values recorded in the solid state. In DFT calculations, bond lengths and angles have been reported only for the major component of the phenyl moiety of **2a** (designated by the suffix “A”). The selected calculated and experimental geometric parameters for **1a** and **2a**,**b** have been tabulated in Table 2, Table 4 and Table 6.

### 2.6. Thermal Gravimetric Analysis (TGA)

**Figure 5:**
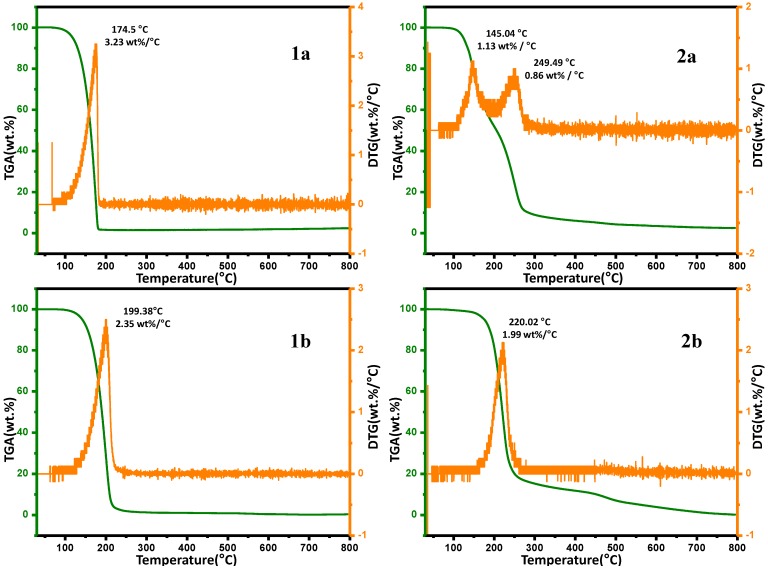
TGA and DTG curves of the synthesized compounds.

The synthesized novel compounds were subjected to thermogravimetric analysis (TGA) to evaluate their thermal stability and degradation patterns (Table 9) and the TGA and DTG patterns are shown in Figure 5. All samples were subjected to analysis in a nitrogen atmosphere in the temperature range from 30 °C to 800 °C with a heating ramp rate of 10 °C per min. It was observed that the different nitrostyrene derivatives displayed different thermal stabilities and degradation patterns. The pyrolysis processes of the materials are characterized by single-stage degradation, with an exception of **2a**, which displays two-stage degradation. Interestingly, it was detected that upon the incorporation of the barbituric acid ring into the styrene, the resulting molecule was slightly more thermally stable than its precursor. The weight loss pattern of all the compounds was found to be different at different intervals with **1a**,**b** displaying a weight loss of 70% and 98% respectively at 200 °C, but at the same temperature the weight loss percentage for the barbituric acid derivatives **2a**,**b** were found to be 48% and 18 % respectively. When a comparison of thermal degradation pattern between **1a** and **2a** was made it was found that **1a** undergoes single stage degradation with decomposition range of 100–190 °C (90 °C), while **2a** displays two stage degradation with decomposition taking place in the range of 105–195 °C (90 °C) and 200–275 °C (75 °C). The degradation temperature range of **1b** and **2b** was found to take place at a 162–262 °C (100 °C) and 125–230 °C (105 °C), respectively. The peak temperature of **2b** (220.02 °C) was higher than that of **1b** (199.38 °C), while the decomposition intensity of **1b** was found to be high with 2.35 wt %/°C compared to 1.99 wt %/°C **2b**. This behaviour can be attributed to the presence of barbituric acid. However, when the temperature was increased further all the compounds followed similar weight loss patterns with varying percentages of residue at the final temperature of 800 °C. From the residual weight values obtained at ~800 °C it can be concluded that there is no significant thermal stability among any of the four compounds tested, however it can be said that thermal stability of the barbituric acid derivative of the styrene molecule is slightly improved compared to that of its precursor. The weight loss percentage at different temperatures is mentioned in the table below and a comparative graphical representation of the compounds is given in Figure 5. A detailed study into the thermal behaviour of the synthesized compounds shall be carried out the thermal kinetics will be reported separately.

**Table 9:** Weight loss percentage at different temperatures for **1a**,**b** and **2a**,**b**.

Temperature	Weight Loss (%)			
	1a	2a	1b	2b
100	0.33	0.63	1.42	0.44
200	70.85	48.06	98.37	18.45
300	98.72	91.07	98.46	84.73
400	98.98	93.95	98.40	88.19
500	99.11	95.67	98.29	92.93
600	99.56	96.47	98.08	96.10
700	99.77	97.14	97.87	98.52
800	99.66	97.47	97.55	99.81

## 3. Experimental Section 

### 3.1. General

All the chemicals were purchased from Aldrich (Riedstraße, Germany), Sigma-Aldrich (St. Louis, MO, USA), Fluka (Buchs, Switzerland), and used without further purification, unless otherwise stated. The structure of **1a** was confirmed by X-ray crystal structure analysis (Bruker AXS GmbH, Karlsruhe, Germany). The crystallographic data for **1a** (CCDC-992700), **2a** (CCDC-992327) and **2b** (CCDC-992701) have been submitted to the Cambridge Crystallographic Data Centre (www.ccdc.cam.ac.uk/data_request/cif). Compound **1a** was prepared from 2,6-dicholorobenzaldehyde and nitromethane as white crystals according to the reported procedure [16]. Colorless block-shaped crystals of the compound suitable for X-ray analysis were formed in isopropanol/heptane at room temperature after 3 days.

### 3.2. 5-(1-(2,6-Dichlorophenyl)-2-nitroethyl)-1,3-dimethylpyrimidine-2,4,6(1H,3H,5H)-trione (**2a**)

According to the reported procedure [16], **2a** was prepared from 1,3-dimethylbarbituric acid (**1a**) and (*E*)-2,6-dichloro-1-(2-nitrovinyl)benzene (**1a**) as white crystals. Colorless block-shaped crystals of the compound suitable for X-ray analysis were formed in CHCl_3_/Et_2_O at room temperature after 2 days.

### 3.3. 5-(1-(2,4-Dichlorophenyl)-2-nitroethyl)-1,3-dimethylpyrimidine-2,4,6(1H,3H,5H)-trione (**2b**)

According to the reported procedure [16], **2b** was prepared from 1,3-dimethylbarbituric acid and (*E*)-2,4-dichloro-1-(2-nitrovinyl)benzene (**1a**) as yellow crystals. Colorless block-shaped crystals of the compound suitable for X-ray analysis were formed in DCM/Pet. ether at room temperature after 1 day.

### 3.4. DFT Calculations

The DFT calculations were performed using the GAMESS package. The input geometry of the synthesized molecules were optimized without imposing any external constraint on the potential energy surfaces generated by the B3LYP/6-311G(d,p) basis set for C, O, N and H atoms. The resulting optimized geometry was used as an input for vibrational frequencies calculations, with inclusion of polarization functions to handle the polar bonds such as N=O, C=O, *etc.*

## 4. Conclusions

In a summary, pyrimidine derivatives **2a**,**b** were prepared starting from nitroalkenes **1a**,**b** using an eco-benign method. The structures for **1a** and **2a**,**b** have been characterized by their single crystal X-ray diffraction analysis. DFT/TGA/IR analyses were performed and discussed. 

## Supplementary Materials

Supplementary materials can be accessed at: http://www.mdpi.com/1420-3049/19/11/17187/s1.

## Supplementary Files

Supplementary File 1
