# Supplementary material for: Synthesis, X-ray Diffraction, Thermogravimetric and DFT Analyses of Pyrimidine Derivatives
Source: Molecules. 2014 Oct 24;19(11):17187–201. doi: 10.3390/molecules191117187 (PMC6271542; doi:10.3390/molecules191117187)
Supplement: Supplementary File 1 [file molecules-19-17187-s001.pdf]

Supplementary Materials

Figure S1. DFT optimized structure of **1a** along with computed data.

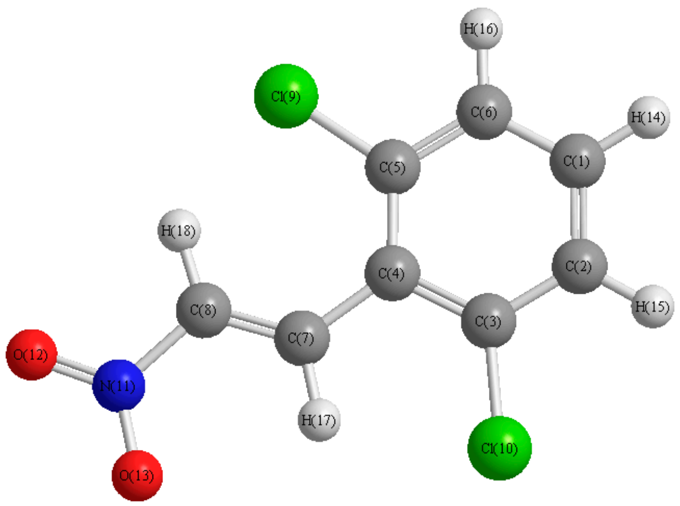

Heat of Formation = 22.075 kcal/mol; Total Energy = −896424.2164 kcal/mol; Zero point energy = 73.289560 kcal/mol; Dipole = (8.061982, −1.892683, −0.000283) 8.281171 Debye.

Table S1. Experimental bond lengths, angles for **1a**.

Atom	Bond Atom	Bond Length	Angle Atom	Angle	Second Angle Atom	Second Angle	Second Angle Type
C(1)							
C(2)	C(1)	1.3852					
C(3)	C(2)	1.4046	C(1)	120.4992			
C(4)	C(3)	1.4243	C(2)	122.2381	C(1)	0.0008	Dihedral
C(5)	C(4)	1.4163	C(3)	115.5409	C(2)	0.0015	Dihedral
C(6)	C(1)	1.3872	C(2)	118.9898	C(3)	−0.0015	Dihedral
C(7)	C(4)	1.4983	C(3)	118.5863	C(5)	125.8729	Pro-S
Cl(9)	C(5)	1.7314	C(4)	124.7209	C(6)	113.6358	Pro-S
Cl(10)	C(3)	1.7405	C(2)	114.3525	C(4)	123.4094	Pro-S
C(8)	C(7)	1.3415	C(4)	130.2898	C(3)	179.9749	Dihedral
N(11)	C(8)	1.4511	C(7)	119.8515	C(4)	−179.9972	Dihedral
O(12)	N(11)	1.2370	C(8)	115.6492	C(7)	−179.9878	Dihedral
O(13)	N(11)	1.2372	C(8)	119.2397	O(12)	125.1111	Pro-R
H(14)	C(1)	1.0871	C(2)	120.5074	C(6)	120.5028	Pro-S
H(15)	C(2)	1.0886	C(1)	119.1681	C(3)	120.3327	Pro-R
H(16)	C(6)	1.0893	C(1)	118.7108	C(5)	120.2005	Pro-S
H(17)	C(7)	1.0888	C(4)	113.6643	C(8)	116.0459	Pro-R
H(18)	C(8)	1.0736	C(7)	130.8355	N(11)	109.3130	Pro-R

Table S2. Experimental transmittance and wavenumber for **1a**.

Transmittance (%)	Wavenumbers
99.44	0.6000
99.74	1.4300
99.88	4.4100
100.00	4.8000
99.99	10.0100
99.46	20.7500
100.00	37.4400
99.94	49.8700
99.18	88.0900
99.78	106.0700
98.21	128.8600
100.00	171.9700
99.26	204.2400
98.87	253.9600
99.86	271.8500
92.67	298.1400
98.62	389.3600
99.98	411.4600
94.80	462.5300
95.62	471.1100
100.00	537.2400
99.82	566.3500
87.17	638.7700
81.08	661.5000
91.94	705.7100
77.88	755.1200
96.00	762.7600
85.07	827.6000
97.71	908.2200
27.50	915.9300
99.79	928.6700
80.72	995.1100
94.82	1009.9600
87.32	1021.9700
79.77	1099.3700
76.02	1134.6000
60.19	1159.4100
67.77	1190.9300
98.92	1193.5300
92.30	1289.2700
84.15	1346.0800
97.32	1375.0300
94.42	1510.1600
98.40	1581.9800
61.84	1600.1900
99.19	1753.4900
95.53	1776.2100
96.54	1844.8300
0.00	1897.0300
99.73	2914.3700
96.48	2965.9200
99.79	3039.6100
98.50	3051.9200
98.85	3068.2100

Figure S2. DFT calculated HOMO and LUMO for **1a**.

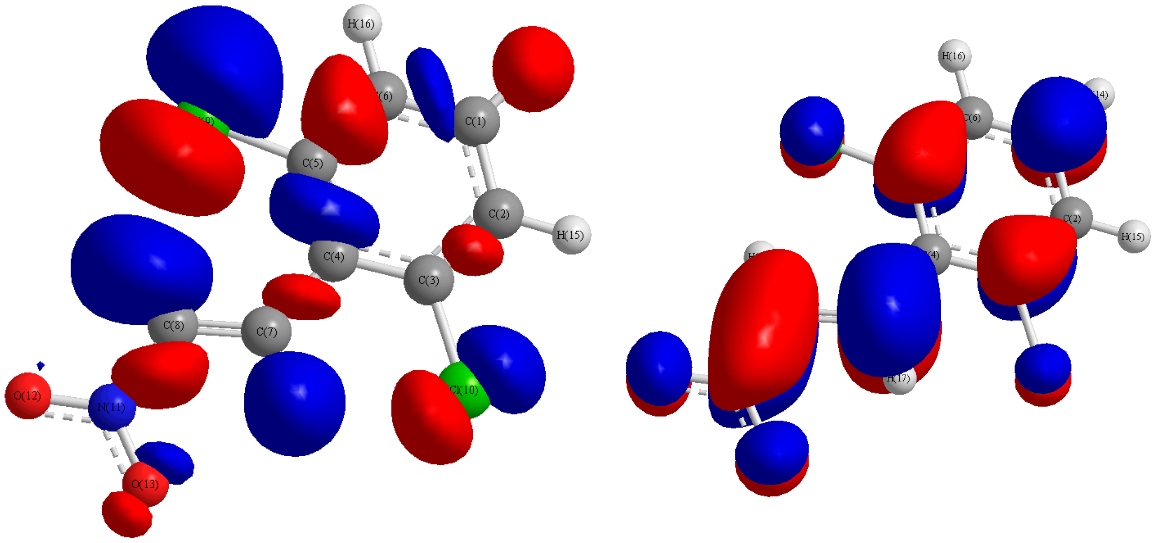

HOMO Energy: −10.019 eV LUMO Energy: −4.320 eV

Figure S3. DFT optimized structure of **2a** along with computed data.

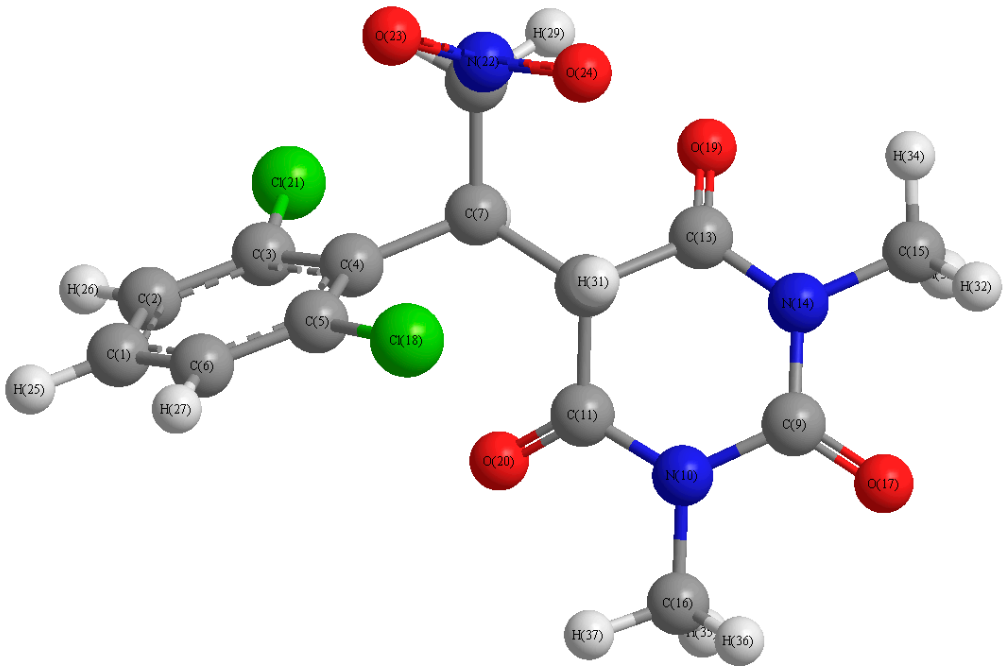

Total Energy = −102495.4693 kcal/mol; Heat of Formation = −99.4723 kcal/mol; Dipole = (2.064781, 2.697171, −1.807120) 3.847562 Debye.

Figure S4. DFT computed HOMO and LUMO for **2a**.

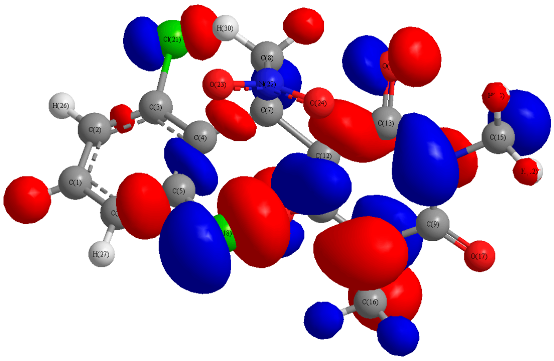

HOMO Energy: −10.813 eV

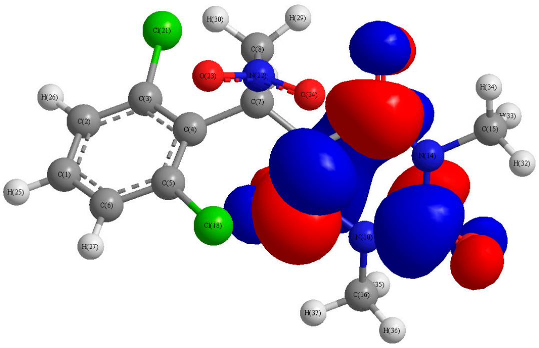

LUMO Energy: −2.383 eV

Table S3. Experimental bond lengths, angles for **2a**.

Atom	Bond Atom	Bond Length	Angle Atom	Angle	Second Angle Atom	Second angle	Second Angle Type
C(1)							
C(2)	C(1)	1.3864					
C(3)	C(2)	1.4015	C(1)	120.3522			
C(4)	C(3)	1.4217	C(2)	122.0382	C(1)	0.5221	Dihedral
C(5)	C(4)	1.4132	C(3)	116.0374	C(2)	−1.3476	Dihedral
C(6)	C(1)	1.3884	C(2)	119.1574	C(3)	0.4247	Dihedral
C(7)	C(4)	1.5485	C(3)	119.1318	C(5)	124.8191	Pro-R
C(12)	C(7)	1.5634	C(4)	118.9024	C(3)	−139.7495	Dihedral
C(11)	C(12)	1.5128	C(7)	113.9543	C(4)	47.2869	Dihedral
C(13)	C(12)	1.5123	C(7)	113.2727	C(11)	106.6992	Pro-S
N(10)	C(11)	1.3874	C(12)	113.0421	C(7)	178.0394	Dihedral
C(9)	N(10)	1.3915	C(11)	122.8908	C(12)	−32.2783	Dihedral
N(14)	C(13)	1.3862	C(12)	113.3505	C(7)	−177.7159	Dihedral
Cl(18)	C(5)	1.7277	C(4)	124.0885	C(6)	114.4806	Pro-R
Cl(21)	C(3)	1.7365	C(2)	115.2511	C(4)	122.7008	Pro-R
C(15)	N(14)	1.4517	C(9)	117.0650	C(13)	117.5998	Pro-R
C(16)	N(10)	1.4516	C(9)	117.0067	C(11)	117.4565	Pro-S
C(8)	C(7)	1.5498	C(4)	109.1372	C(12)	115.7561	Pro-R
O(17)	C(9)	1.2323	N(10)	121.7606	N(14)	121.7552	Pro-R
O(19)	C(13)	1.2279	C(12)	123.0484	N(14)	123.5988	Pro-R
O(20)	C(11)	1.2275	N(10)	123.3054	C(12)	123.6516	Pro-R
N(22)	C(8)	1.4968	C(7)	111.7613	C(4)	77.9778	Dihedral
O(23)	N(22)	1.2363	C(8)	117.2469	C(7)	−101.8952	Dihedral
O(24)	N(22)	1.2377	C(8)	116.9998	O(23)	125.7525	Pro-R
H(25)	C(1)	1.0868	C(2)	120.4529	C(6)	120.3881	Pro-S
H(26)	C(2)	1.0881	C(1)	119.2359	C(3)	120.4115	Pro-R
H(27)	C(6)	1.0889	C(1)	118.8242	C(5)	120.2011	Pro-S
H(31)	C(12)	1.0927	C(7)	110.2297	C(11)	106.9205	Pro-R
H(28)	C(7)	1.0980	C(4)	105.1256	C(8)	104.4393	Pro-R
H(29)	C(8)	1.0922	C(7)	113.5886	N(22)	105.8185	Pro-S
H(30)	C(8)	1.0963	C(7)	112.1473	N(22)	106.4889	Pro-R
H(32)	C(15)	1.0943	N(14)	110.3782	C(9)	−44.3751	Dihedral
H(33)	C(15)	1.0948	N(14)	108.6067	H(32)	108.9451	Pro-S
H(34)	C(15)	1.0941	N(14)	110.8285	H(32)	108.9144	Pro-R
H(35)	C(16)	1.0948	N(10)	108.6479	C(9)	−74.7931	Dihedral
H(36)	C(16)	1.0942	N(10)	110.3827	H(35)	108.9658	Pro-S
H(37)	C(16)	1.0942	N(10)	110.7622	H(35)	109.1393	Pro-R

Table S4. Experimental transmittance and wavenumber for **2a**.

Transmittance (%)	Wavenumbers
99.98	5.9800
100.00	7.7300
99.98	11.0900
99.96	13.5700
100.00	17.2900
99.79	19.1400
99.72	27.8200
99.75	31.5900
99.49	34.2100
99.72	46.5400
99.95	46.7100
97.59	50.1100
99.64	65.3300
95.75	68.4300
99.84	81.0600
99.93	90.5300
99.19	101.1700
99.65	118.9400
99.04	156.3700
98.27	166.8700
99.99	181.6700
99.81	184.3600
98.59	193.7700
98.52	200.6100
99.11	250.5400
98.27	275.8700
99.63	286.2400
94.83	344.8000
95.99	367.8200
93.17	373.7400
99.32	379.4600
98.47	394.2000
99.29	407.2600
97.50	409.1400
97.46	432.6800
96.21	461.5700
90.52	471.2400
99.08	491.7600
84.97	523.9700
99.62	535.1000
91.62	591.7700
96.39	604.7200
84.17	611.3800
47.99	629.4200

Table S4. *Cont.*

Transmittance (%)	Wavenumbers
97.84	633.5200
99.23	662.4500
85.66	688.7100
71.88	737.3900
96.39	761.2300
49.43	787.5100
86.57	818.4100
92.18	840.6400
95.78	861.5100
85.35	891.0800
99.95	932.8400
87.04	935.3500
82.81	948.4000
85.78	963.1200
99.26	985.5100
99.29	992.5100
98.82	1012.7700
92.46	1024.6000
95.70	1032.0800
90.42	1047.2900
98.60	1057.2100
97.48	1083.7900
89.61	1099.5500
66.34	1128.1300
81.74	1142.1900
85.35	1172.0400
78.91	1175.5100
98.26	1183.4700
80.45	1200.0600
86.40	1216.2400
86.05	1252.2900
88.30	1274.6800
71.39	1281.6100
79.38	1293.0800
87.16	1324.4000
57.39	1332.4800
71.41	1348.6900
84.12	1361.6200
85.29	1365.6000
45.97	1372.3200
91.32	1373.7200
96.96	1379.7800
88.34	1385.6200
89.95	1396.8900
91.71	1403.3000

Table S4. *Cont.*

Transmittance (%)	Wavenumbers
94.35	1514.8300
95.58	1581.8300
67.64	1604.3400
99.32	1760.8000
96.00	1781.9300
0.00	1894.6100
96.35	1899.8500
90.43	1929.9300
95.28	1971.2700
98.66	2785.9600
99.97	2808.9400
99.45	2888.3300
99.48	2972.5400
99.33	3036.0600
98.86	3050.0200
99.54	3051.6700
99.05	3059.3500
98.98	3064.7200
99.30	3064.9600
99.16	3069.1800
99.93	3130.1100
99.96	3141.1500

Figure S5. DFT optimized structure of **2b** along with computed data.

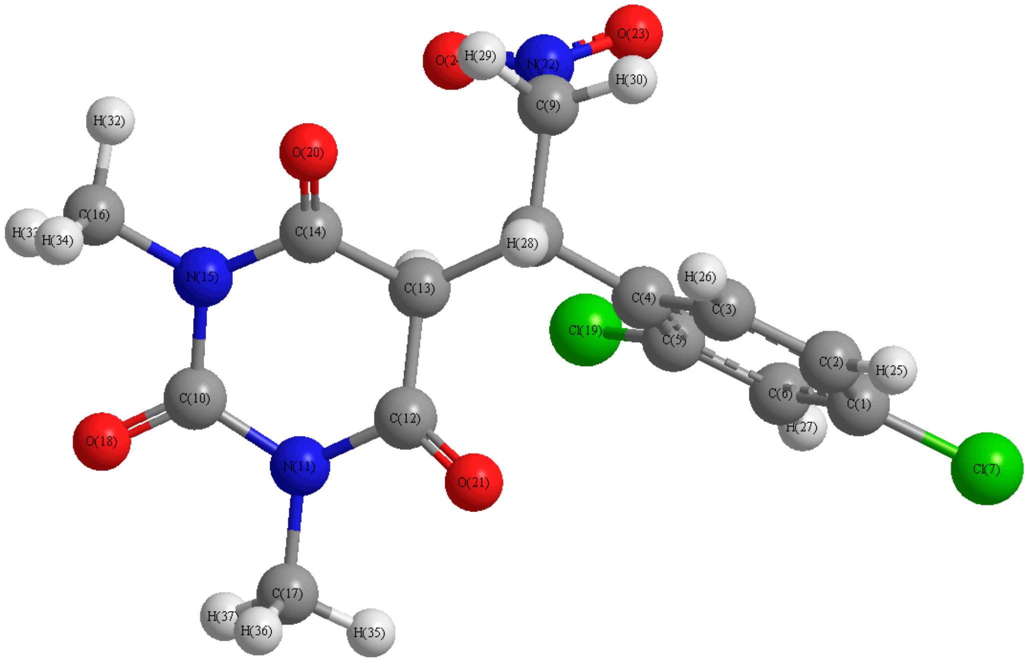

Total Energy = −1244859.4433 kcal/mol; Heat of Formation = −100.9025 kcal/mol; Dipole = (0.034664, 1.212610, −3.933575) 4.116386 Debye.

Figure S6. DFT computed HOMO and LUMO for **2b**.

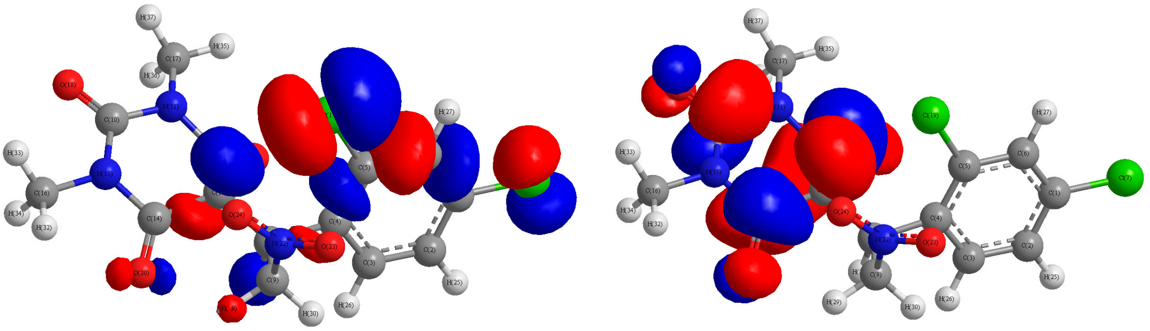

HOMO Energy: −10.575 eV   LUMO Energy: −2.278 eV

Table S5. Experimental bond lengths, angles for **2b**.

Atom	Bond Atom	Bond Length	Angle Atom	Angle	Second Angle Atom	Second angle	Second Angle Type
C(1)							
C(2)	C(1)	1.3873					
C(3)	C(2)	1.3967	C(1)	119.6476			
C(4)	C(3)	1.4153	C(2)	121.7288	C(1)	0.2557	Dihedral
C(5)	C(4)	1.4090	C(3)	117.2212	C(2)	−0.3285	Dihedral
C(6)	C(1)	1.3898	C(2)	120.1762	C(3)	−0.0114	Dihedral
Cl(7)	C(1)	1.7195	C(2)	119.8773	C(6)	119.9464	Pro-S
C(8)	C(4)	1.5424	C(3)	116.0661	C(5)	126.7037	Pro-R
C(13)	C(8)	1.5593	C(4)	117.8002	C(3)	−135.9436	Dihedral
C(12)	C(13)	1.5125	C(8)	113.9155	C(4)	50.0563	Dihedral
C(14)	C(13)	1.5104	C(8)	113.4334	C(12)	106.5256	Pro-S
N(11)	C(12)	1.3881	C(13)	113.0470	C(8)	177.8067	Dihedral
C(10)	N(11)	1.3925	C(12)	123.0628	C(13)	−30.7868	Dihedral
N(15)	C(14)	1.3855	C(13)	113.0492	C(8)	−179.2941	Dihedral
Cl(19)	C(5)	1.7247	C(4)	123.7093	C(6)	115.3937	Pro-R
C(16)	N(15)	1.4513	C(10)	117.0595	C(14)	117.6528	Pro-R
C(17)	N(11)	1.4515	C(10)	116.9594	C(12)	117.4665	Pro-S
C(9)	C(8)	1.5484	C(4)	109.1486	C(13)	116.5427	Pro-R
O(18)	C(10)	1.2325	N(11)	121.7820	N(15)	121.7421	Pro-R
O(20)	C(14)	1.2278	C(13)	123.1300	N(15)	123.8208	Pro-S
O(21)	C(12)	1.2276	N(11)	123.3190	C(13)	123.6338	Pro-S
N(22)	C(9)	1.4962	C(8)	112.1510	C(4)	78.8856	Dihedral
O(23)	N(22)	1.2362	C(9)	117.2089	C(8)	−105.8715	Dihedral
O(24)	N(22)	1.2378	C(9)	117.0208	O(23)	125.7643	Pro-R
H(25)	C(2)	1.0872	C(1)	120.5061	C(3)	119.8459	Pro-S
H(26)	C(3)	1.0898	C(2)	117.6896	C(4)	120.5779	Pro-S
H(27)	C(6)	1.0876	C(1)	119.5477	C(5)	120.1232	Pro-S
H(31)	C(13)	1.0929	C(8)	110.1325	C(12)	106.6973	Pro-R
H(28)	C(8)	1.1026	C(4)	104.3839	C(9)	103.7494	Pro-R
H(29)	C(9)	1.0921	C(8)	113.5301	N(22)	105.8036	Pro-S
H(30)	C(9)	1.0968	C(8)	111.6415	N(22)	106.7884	Pro-R
H(32)	C(16)	1.0940	N(15)	110.7198	C(10)	−165.8870	Dihedral
H(33)	C(16)	1.0943	N(15)	110.2854	H(32)	108.9633	Pro-S
H(34)	C(16)	1.0947	N(15)	108.6865	H(32)	109.1871	Pro-R
H(35)	C(17)	1.0942	N(11)	110.8294	C(10)	165.3616	Dihedral
H(36)	C(17)	1.0950	N(11)	108.5925	H(35)	109.1156	Pro-S
H(37)	C(17)	1.0943	N(11)	110.4156	H(35)	108.9306	Pro-R

Table S6. Experimental transmittance and wavenumber for **2b**.

Transmittance (%)	Wavenumbers
99.87	5.1100
100.00	8.4700
99.91	10.5600
99.95	16.3500
99.88	19.5500
99.73	20.0300
99.62	22.7900
99.82	30.7000
99.75	32.8000
99.89	38.8200
99.53	48.2000
98.07	52.4500
99.63	56.7700
95.71	70.4900
99.92	93.5900
99.16	101.1000
99.63	102.7400
99.92	108.6900
99.15	156.0300
99.32	162.0900
98.79	170.7800
99.74	189.2400
99.44	193.5100
99.01	201.2900
98.25	218.5600
97.85	268.9100
98.07	294.9900
94.21	342.1700
99.39	369.2500
92.71	372.4400
99.28	377.5000
99.62	389.4900
99.05	403.2500
99.91	406.6300
99.18	422.2400
86.57	458.4600
99.21	462.6100
95.69	471.3100
91.31	519.9400
88.63	566.4700
92.84	588.6300
90.66	603.8800
86.83	608.4900
46.12	628.1200
98.71	642.1000

Table S6. *Cont.*

Transmittance (%)	Wavenumbers
99.94	672.3300
87.83	689.3100
82.30	721.5900
85.92	729.5300
76.98	764.6800
79.20	839.5800
80.68	860.1300
96.07	863.7300
87.80	893.1100
85.80	934.9300
91.19	939.8600
83.27	946.2200
88.68	960.2100
99.31	983.7400
99.02	991.5200
99.14	992.9200
90.48	1022.0800
95.30	1031.1800
89.84	1046.1900
95.31	1048.7500
79.09	1068.9000
98.55	1090.9700
89.48	1113.3900
64.89	1127.9300
73.02	1149.8800
81.03	1175.9500
97.00	1181.9300
77.17	1189.9400
97.88	1209.1100
86.66	1242.6500
97.85	1270.7600
61.91	1281.7600
80.47	1293.7100
95.72	1319.3400
52.82	1332.4600
66.66	1351.0100
84.03	1361.8200
85.89	1365.4000
50.33	1370.2600
90.41	1376.5900
93.44	1380.1600
90.99	1394.1200
89.17	1397.4900
92.78	1403.2800
97.25	1511.2400

Table S6. *Cont.*

Transmittance (%)	Wavenumbers
94.01	1583.7900
67.98	1603.0600
99.01	1762.6000
98.80	1786.7300
100.00	1894.7500
96.99	1900.7300
90.44	1930.2500
95.13	1970.7300
98.66	2783.6200
99.99	2819.9200
99.52	2900.0200
99.59	2980.6500
97.83	3037.1500
99.57	3046.0600
99.62	3047.3100
99.02	3054.3700
99.32	3058.0500
99.18	3068.9500
98.69	3074.5400
99.92	3124.0200
99.95	3138.5100
